# High-Throughput LC-ESI-MS/MS Metabolomics Approach Reveals Regulation of Metabolites Related to Diverse Functions in Mature Fruit of Grafted Watermelon

**DOI:** 10.3390/biom11050628

**Published:** 2021-04-23

**Authors:** Ali Aslam, Shengjie Zhao, Xuqiang Lu, Nan He, Hongju Zhu, Aman Ullah Malik, Muhammad Azam, Wenge Liu

**Affiliations:** 1 Zhengzhou Fruit Research Institute, Chinese Academy of Agricultural Sciences, Zhengzhou 450009, China; mianaliaslam538@gmail.com (A.A.); zhaoshengjie@caas.cn (S.Z.); luxuqiang@caas.cn (X.L.); henan@caas.cn (N.H.); zhuhongju@caas.cn (H.Z.); 2 Institute of Horticultural Sciences, University of Agriculture, Faisalabad 38000, Punjab, Pakistan; malikaman1@uaf.edu.pk (A.U.M.); azam32jb@yahoo.com (M.A.)

**Keywords:** watermelon, metabolites, grafting

## Abstract

Grafting has been reported as a factor regulating the metabolome of a plant. Therefore, a comprehensive metabolic profile and comparative analysis of metabolites were conducted from fully mature fruit of pumpkin-grafted watermelon (PGW) and a self-rooted watermelon (SRW). Widely targeted LC-ESI-MS/MS metabolomics approach facilitated the simultaneous identification and quantification of 339 metabolites across PGW and SRW. Regardless of grafting, delta-aminolevulinic acid hydrochloride, sucrose, mannose-6-phosphate (carbohydrates), homocystine, 2-phenylglycine, s-adenosyl-L-homocysteine (amino acids and derivatives), malic, azelaic, H-butanoic acid ethyl ester-hexoside isomer 1, (organic acids), MAG (18:3) isomer1, LysoPC 16:0, LysoPC 18:2 2n isomer (lipids) p-coumaric acid, piperidine, and salicylic acid-o-glycoside (secondary metabolites) were among the dominant metabolite. Dulcitol, mono-, and disaccharide sugars were higher in PGW, while polysaccharides showed complex behavior. In PGW, most aromatic and nitrogen-rich amino acids accumulated greater than 1.5- and 1-fold, respectively. Intermediates of the tricarboxylic acid cycle (TCA), stress-related metabolites, vitamin B5, and several flavonoids were significantly more abundant in PGW. Most lipids were also significantly higher in grafted watermelon. This is the first report providing a comprehensive picture of watermelon metabolic profile and changes induced by grafting. Hence, the untargeted high-throughput LC-ESI-MS/MS metabolomics approach could be suitable to provide significant differences in metabolite contents between grafted and ungrafted plants.

## 1. Introduction

Watermelon (*Citrullus lanatus* L.) is one of the most economically important horticultural crops, with 83.7% production in Asia and 9.5% production globally out of total vegetable production. According to 2017 data, China is the leading producer of watermelon with more than half of the world’s production, and around 20% of crops come from grafted plants (http://faostat.fao.org accessed on 15 December 2019). Watermelon is a rich source of metabolites such as vitamins, minerals, fiber, antioxidants, organic acids, sugars, and amino acids [1,2,3]. Plant primary and secondary metabolites play an integral role in regulating the plant’s growth and development, pigmentation of flowers and fruits, flavor, defense mechanisms against diseases, and tolerance to unfavorable environmental conditions [4,5]. Secondary metabolites are distributed widely among plants and are involved in multiple biological functions, including plant protection against U.V light, pathogen attack, and male sterility [6]. In plants, amino acids, nucleotides, fatty acids, carbohydrates, and organic acids are mainly included in primary metabolites. In contrast, phenolic acids, flavonoids, polyamines, alkaloids, phytohormones and vitamins are included in secondary metabolites [7,8].

Information regarding extensive metabolite profiling is inadequate not only in watermelon as well as in other crops. However, few reports on the limited number of metabolites are available. For example, Kader (2008) states that watermelon’s organoleptic quality is principally dependent on amino acids, sugars, and organic acids. The main sugars of watermelon are sucrose, glucose, and fructose. Sucrose alone accounts for 70% of watermelon reducing sugars [9,10]. In the acid profile, citric, oxalic, and malic acids are predominant. Based on limited metabolite profiling [11] concluded that malic and oxalic acids are the main acids present in watermelon flesh. Different amino acid offers different taste, and they represent a large portion of watermelon’s metabolic profile. Amino acids take part in the production of primary and secondary metabolites such as aroma compounds and vitamins [12,13]. Recently, it was reported the metabolites vary significantly among and within *Citrullus* species. A total of 431 metabolic features were detected, and metabolite-related to phenols showed significant divergence in different flesh color watermelons [14].

Genotype, environmental conditions, cultural practices, and grafting play a key role in regulating the fruit chemical composition, primary and secondary metabolites [15,16,17,18]. Grafting is an innovative and accessible technique that has been used in continuous cropping systems around the world. Grafting increases yield and production and fulfills the needs of the growing global population [19]. Previously, grafting has been used for vegetable production to inhibit soil pathogens’ effects [20]. It has gained incredible significance in the global horticulture industry, and demand has increased dramatically due to high yield and increased resistance against stresses, pests, and soil-borne diseases [21]. Plant growth and development are seriously affected by these everyday environmental stresses. Grafting increases vegetable production [22,23,24], nutrient uptake, eliminate the effects of salinity, toxicity and enhance fruit quality [25,26,27,28,29]. Grafting is an environmentally friendly technique for reducing the loss due to salinity, biotic, and abiotic stresses by regulating primary and secondary metabolites.

In many fruit crops, rootstock has been reported to induce reprogramming of metabolic pathways associated with plants’ divergent functions. Comparative analysis of the citrus scion grafted on to sour orange and rough lemon rootstocks showed significant modification in the metabolic pathways. Sour orange was responsible for increasing the polyphenolics and limonoidaglycones contents, whereas lemon rootstock successfully enhanced the B-complex and C vitamins, volatile aromatic compounds, and few phenols [30]. Similar results were induced by grafting in cherry, where grafting reshaped the sugar composition, increased the phenolics and anthocyanins [31]. Metabolism of compounds such as volatiles, vitamins, sugars, organic acids, and amino acids distinctively amended following the grafting. Grafting onto interspecific rootstock produced sweeter and quality melons due to changes in carbohydrate metabolism that increased carbohydrates metabolic enzyme activities and enhanced the sugars contents [32].The primary and secondary metabolic pathway of amino acids, alcohols, sugars and organic acid changed drastically upon grafting in cucumber [33]. In tea, grafting affected the flavonoid, theanine, and caffeine pathways. Higher contents of total catechins and caffeine were found in grafted plants leaves, whereas total amino acids were more in ungrafted plants leaves [34].

Likewise, morphological attributes and a limited number of fruit quality parameters, including major sugars, acids, and TSS, were influenced by grafting in many crops [35]. Grafting in watermelon influences phytochemicals such as organic acids, lycopene, carotenoids, and ascorbic acid by mediating the transcript level of genes or translocating metabolites [36,37]. Scion grafting in mandarin significantly affected the metabolic content of juice, and several metabolites were different between the sap of scion and rootstock [38]. Metanalysis reported by [39] showed improvement in fruit lycopene contents, pH, firmness, soluble solids, Vitamin C, and taste resulting from the grating in tomato. Grafting caused a reduction in the total yield of amino acids in muskmelon fruit at maturity affecting primary metabolism [40]. In contrast, grafting in grapes increased the amino acid contents of valine, glutamine, arginine, isoleucine, serine, threonine, and leucine [41]. Cucurbita rootstocks in watermelon induced transcriptional reprogramming of citrulline genes, thus influencing watermelon’s citrulline contents [42].

Several cucurbit rootstocks such as *Benincasa hispida*, *Cucurbita maxima*, *Luffa cylindrica*, *C. ficifolia*, *Lagenaria siceraria*, *C. argyrosperma*, and *C. moschata* are currently being used in watermelon production [43,44,45]. However, the use of pumpkin rootstock to improve fruit nutritional quality and sensory parameters such as taste and pulp texture is more common in watermelon production [46,47,48,49].

Previous studies mainly focused on the effect of grafting on major sugars, few metabolites, pH, titrability, acidity, and volatile compounds, survival rates, yields, effects on disease resistance. To date, no study has reported comprehensive metabolite profiling and comparative analysis of metabolites from fully mature fruit of grafted and ungrafted watermelon. In the present study, watermelon scion Zhongyu No. 1 was grafted onto pumpkin rootstock Xi Jia Qiang Sheng (*Cucurbita* sp.), and ungrafted (self-rooted watermelon) watermelon was used as control.

## 2. Materials and Methods

The plants were grown in a plastic greenhouse from March to July 2018 in Xinxiang city, China. Watermelon (*C. lanatus* (*Thunb*.) Matsum. and Nakai var. *lanatus,* cultivar Zhongyu No. 1) is a diploid mini watermelon characterized by a green skin with dark green stripes and yellow flesh was grafted onto pumpkin Xi Jia Qiang Sheng (*Cucurbita* sp.), watermelon that was not grafted served as control. All plant materials were obtained from the Laboratory of Polyploidy Watermelon Breeding, Zhengzhou Fruit Research Institute, Chinese Academy of Agricultural Sciences. Seeds were sown in plastic trays (32 cell tray) containing peat moss, and top insertion grafting was carried out with 15 days old scion according to a previously reported method [20]. In order to achieve the diameter of the same size, scion stock was sown five days before rootstock seeds. Plants were shifted to a green plastic house, on the appearance of the third true leaf. Rows were 150 cm apart, and plant-to-plant distance was maintained at 50 cm. A randomized complete block design with three replications was used. Each plot consisted of 40 plants in a single row. Plants were trained to a single stem by clipping offside branches and were supported with rope. Only one fruit was allowed to develop on each plant. Standard field management procedures such as pest and disease control, weeding, fertilizer application, and irrigation were implemented during the growing season. At the onset of flowering, the female flowers were manually pollinated on the same day, and tagging was performed to record the number of days after pollination (DAP). Three healthy watermelon fruits, uniform in shape and size from three independent plants at the mature stage (34 days after pollination) in each treatment were harvested (Figure S1). Watermelons were harvested early morning before sunrise and shifted to the laboratory on ice. In the lab, watermelons were longitudinally cut into two pieces using the sterilized knife. In total, six fruit flesh samples were collected from the heart area (center) of the watermelon, then promptly frozen in liquid nitrogen and stored at −80 °C for extraction of metabolites.

### 2.1. Chemicals

All solvents such as methanol, acetonitrile, and methanol (Merck, Darmstadt, Germany) were HPLC-grade. Double deionized water with a Milli QULTRA purification system (Millipore, Vimodrone, Italy) was used for solutions. All original standards were acquired from Sigma-Aldrich, St. Louis, MO, USA (www.sigmaaldrich.com/united-states.html accessed on 13 August 2018). Methanol and deionized water were used as the respective solvents for preparing stock solutions and were kept at −20 °C for downstream analysis.

### 2.2. Sample Preparation and Extraction Chemicals

The freeze-dried flesh of watermelon fruit was crushed using a mixer mill (MM 400, Retsch) with a zirconia bead for 1.5 min at 30 Hz. A total of 100 mg powder was weighted and extracted overnight at 4 °C with 1 mL 70% aqueous methanol. Following centrifugation at 10,000× *g* for 10 min, the extracts were absorbed (CNWBOND Carbon-GCB SPE Cartridge, 250 mg, 3 mL; ANPEL, Shanghai, China, www.anpel.com.cn/cnw accessed on 12 August 2018) and filtrated (SCAA-104, 0.22 μm pore size; ANPEL, Shanghai, China, http://www.anpel.com.cn/ accessed on 12 August 2018) before LC-MS analysis.

### 2.3. Analytical Condition of LC-MS/MS

Analyses of sample extracts were carried out using a liquid chromatography-electrospray ionization mass spectrometry (LC-ESI-MS/MS) system (HPLC, Shim-pack UFLC SHIMADZU CBM30A system, www.shimadzu.com.cn/ accessed on 12 August 2018); MS, Applied Biosystems 6500 Q TRAP, (www.appliedbiosystems.com.cn accessed on 12 August 2018) at Metware (Wuhan, China). The LC-MS/MS system operated under the following conditions: HPLC: column, Waters ACQUITY UPLC HSS T3 C18 (1.8 μm, 2.1 mm * 100 mm); solvent system, water (0.04% acetic acid): acetonitrile (0.04% acetic acid); gradient program, 95:5 *v*/*v* at 0 min, 5:95 *v*/*v* at 11.0 min, 95:5 *v*/*v* at 12.0 min, 95:5 *v*/*v* at 12.1 min, 95:5 *v*/*v* at 15.0 min; flow rate, 0.40 mL/min; temperature, 40 °C; injection volume: 2 μL. LIT and triple quadrupole (QQQ) scans were acquired on a triple quadrupole-linear ion trap mass spectrometer (Q TRAP), API 6500 Q TRAP LC/MS/MS System, supplied with an ESI Turbo Ion-Spray interface, running in a positive ion mode and controlled by Analyst 1.6.3 software (AB Sciex). The ESI source operation conditions were set as ion source, turbo spray; source temperature 500 °C; ion-spray voltage (IS) 5500 V; ion source gas I (GSI), gas II(GSII), curtain gas was set at 65, 60, and 25.0 psi, respectively; the collision gas was high. In QQQ and LIT modes, 10 and 100 μmol/L polypropylene glycol solutions were used to perform the tuning of the instrument and mass calibration, respectively. QQQ scans were acquired as MRM experiments with collision gas (nitrogen) set to 5 psi. DP and CE for individual MRM transitions were performed with further declustering potential (DP) and collision energy (CE), optimization. During the elution of metabolites in the specific period, MRM transitions corresponding to each metabolite were observed within that period [50].

A quality control check was executed as an effective measure to ensure data reliability. For this reason, the QC sample was prepared by the mixture of sample extracts and inserted into every two samples to monitor the changes in repeated analyses. Qualitative and quantitative analyses of metabolites were undertaken using the methods of [51]. The qualitative analysis of primary and secondary mass spectrometry data was performed based on the self-built database MWDB (Metware Biotechnology Co., Ltd., Wuhan, China) and the publicly available metabolite databases. The interference from isotope signals; repeated signals of K^+^, Na^+^, NH4^+^ ions, and fragment ions derived from other larger molecules were eliminated during identification. Metabolite structure analysis was obtained by referencing existing mass spectrometry databases such as MassBank (http://www.massbank.jp accessed on 7 January 2019), KNAPSAcK (http://kanaya.naist.jp/KNApSAcK accessed on 8 January 2019), and METLIN (http://metlin.scripps.edu/index.php accessed on 8 January 2019).

### 2.4. Statistical Analysis

Important metabolic pathways were drawn using the Kyoto Encyclopedia of Genes and Genomes (KEGG) database as a reference (https://www.genome.jp/kegg/kegg1.html accessed on 4 November 2019). Pathway illustrations and bar graphs were created using Excel, 2007. Analysis of variance was performed and tested for statistical significance using Statistics 8.1 (Statistics Software, Tallahassee, FL, USA). Tukey’s honest significant difference test was used for the comparison among treatments means *p* < 0.05. Fold change represents the ratio of the mean of each metabolite in two treatments. Fold change was calculated by using PGW/SRW formulas.

## 3. Results

### 3.1. Metabolome Comparison between Pumpkin-Grafted (PGW) and Self-Rooted Watermelon (SRW)

**Figure 1:**
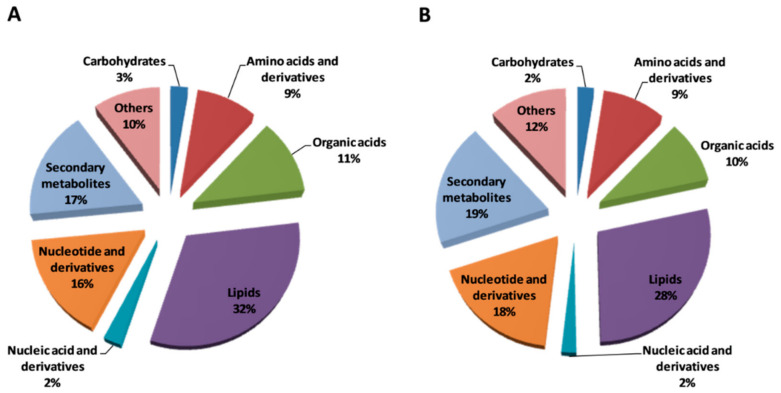
Functional classification of metabolites (**A**) pumpkin-grafted watermelon (PGW) (**B**) self-rooted watermelon (SRW).

To compare the metabolomics profiles of fully mature fruit from pumpkin-grafted watermelon (PGW) and a self-rooted watermelon (SRW) were analyzed based on LC-ESI-MS/MS high-throughput technology. In total, 339 metabolites were detected, of which 304 (90%) metabolites were classified into different classes while 33 (10%) were grouped into others. The percentage share of metabolite classes between treatments showed little variation, but the share percentage of different classes in the same treatment was markedly different. Of which lipids showing the highest percentage (28–32%), followed by secondary metabolites (17–19%), nucleotides and derivatives (16–18%), others (10–12%), organic acids (10–11%), amino acid and derivatives (9%), carbohydrates (2–3%), and nucleic acid and derivatives (2%) (Figure 1).

### 3.2. Major Metabolites

In total, 339 metabolites were identified with either high or low abundance between PGW and SRW. In each class, dominant metabolites were calculated regardless of grafting. It was found that delta-aminolevulinic acid hydrochloride, sucrose, mannose-6- phosphate (carbohydrates), homocysteine, 2-phenylglycine, s-adenosyl-L-homocysteine (amino acids and derivatives), malic, azelaic, H-butanoic acid ethylester-hexoside isomer 1, (organic acids MAG (18:3) isomer1, LysoPC 16:0, LysoPC 18:2 (2n isomer) (lipids), trans-zeatin N-glucoside, salicylic acid, ABA (hormones), pyridoxine O-glucoside, D-pantothenic acid, pyridoxine (vitamins), piperidine, trigonelline, isoquinoline (alkaloids), sorbitol (polyols), triterpenes-cucurbitacin (terpene), salicylic acid-O-glycoside, cinnamic acid, p-coumaric acid (phenylpropanoids,) 3-indoleacetonitrile, indole, 5-hydroxytryptophol (indole derivatives), nicotinic acid-hexoside (nicotinic acid derivatives), uracil, 1-methyladenosine, 2-methyladenosine (nucleic acid and derivative), adenosine, 6-methylmercaptopurine, guanosine (nucleotide and its derivates), genistin (isoflavone), agmatine (phenolamide), 4-pyridoxic acid (pyridine derivatives), campesterol, soyasapogenol B, cycloartenol (sterol), N-acetyl-5-hydroxytryptamine (tryptamine derivatives), aminopurine, viscumamide, trans-2-hexenal (others) were among the dominant metabolites. (Supplementary Materials).

### 3.3. Carbon Metabolism

**Figure 2:**
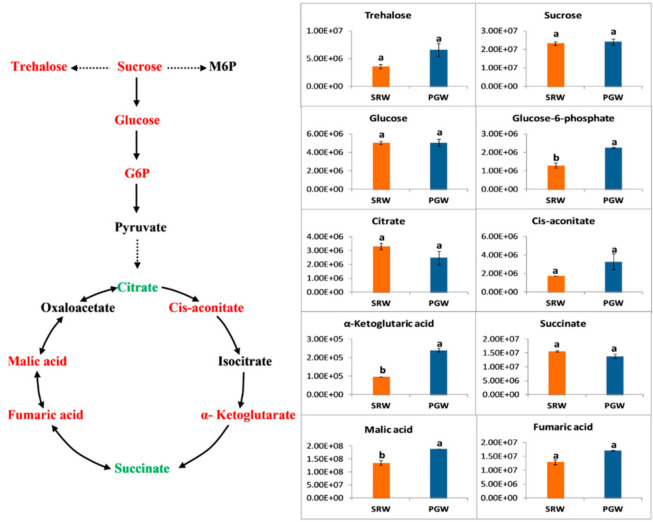
*Y*-axis shows changes in relative contents of metabolites associated with carbon metabolism between mature fruit of pumpkin-grafted watermelon (PGW) and self-rooted watermelon (SRW). Red or green color shows the higher content of metabolites in PGW or SRW, respectively. Vertical bars represent standard error among three independent replicates. Values are the mean ± SE of three replicates. Different lowercase letters indicate significant differences at *p* < 0.05. M6P, maltose-6-phosphate; G6P, glucose-6-phosphate.

Carbohydrates are metabolized during glycolysis and tricarboxylic acid (TCA) cycles to provide a carbon skeleton for other metabolites. Glucose is converted into pyruvate through series of enzymatic reactions in the glycolysis pathway, which further produce either acetyl-CoA or OAA through oxidation or carboxylation, respectively. TCA cycle starts with the condensation of acetyl-CoA and OAA to yield citrate, followed by conversion of citrate into iso-citrate through aconitase enzyme. In the next step, iso-citrate is dehydrogenized into α-ketoglutarate, which is further converted into succinyl-CoA by the α-ketoglutarate dehydrogenase enzyme. Oxaloacetate is produced from succinyl-CoA via the synthesis of succinate, fumarate, and malate [52]. Clear differences among the metabolites of the gluconeogenesis pathway, sugar metabolism, and tricarboxylic acid cycle (TCA) were observed on comparing mature fruit of PGW and SRW (Figure 2). Glucose-6-phosphate, an intermediate of gluconeogenesis pathway, was significantly (*p* < 0.05) greater by 1.75-fold in PGW. Relative contents of two trehalose and sucrose were 1.03- and 1.82-folds higher in PGW, respectively, but the difference was not significant statistically. Glucose is an essential sugar of watermelon, which was almost similar in PGW and SRW fruits. Intermediates of the tricarboxylic acid cycle such as citrate, cis-aconitate, α-ketoglutaric acid, succinate, malic acid, and fumaric acid changed in response to grafting. Malic and fumaric acid showed a 1.40- (*p* < 0.05) and 1.32-folds (*p* > 0.05) increase in PGW, while the relative abundance of citrate and succinate were higher by 0.75- and 0.88-folds in SRW. Notably, α-ketoglutaric acid displayed 2.51-folds increase (*p* < 0.05) in PGW, while the relative content of cis-aconitate was 1.87-folds (*p* > 0.05) greater in PGW.

### 3.4. Carbohydrate Metabolism

**Figure 3:**
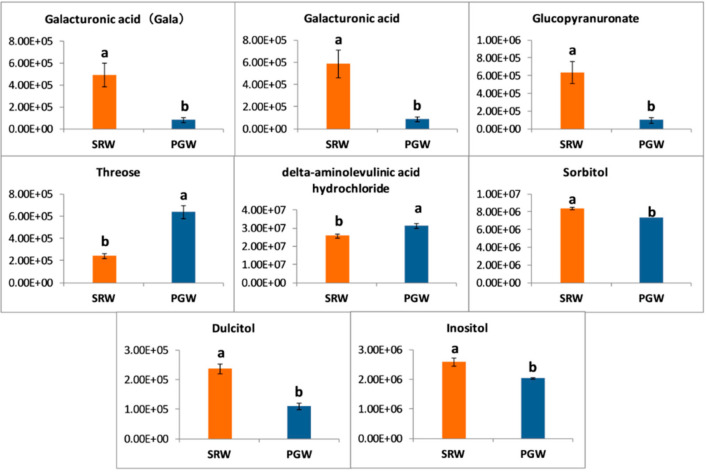
*Y*-axis shows changes in relative contents of carbohydrates between mature fruit of pumpkin-grafted watermelon (PGW) and self-rooted watermelon (SRW). Vertical bars represent standard error among three independent replicates. Data are the mean ± SE of three replicates. Different lowercase letters indicate significant differences at *p* < 0.05.

Among the carbohydrates, threose and delta-aminolevulinic acid hydrochloride exhibited 2.63- and 1.21-folds higher contents (*p* < 0.05) in mature fruit of PGW. In contrast, galacturonic acid (gala), galacturonic acid, and glucopyranuronate showed a significant (*p* < 0.05) decline of 0.17-, 0.15-, and 0.16-folds as a consequence of grafting in PGW, respectively. Polyols are a distinct group of carbohydrates with their role in fruit quality and texture. Fruits from the grafted plant contained a significantly (*p* < 0.05) higher amount of dulcitol, but the relative levels of inositol and sorbitol were more (*p* > 0.05) in ungrafted watermelon (Figure 3).

### 3.5. Arginine Metabolism

**Figure 4:**
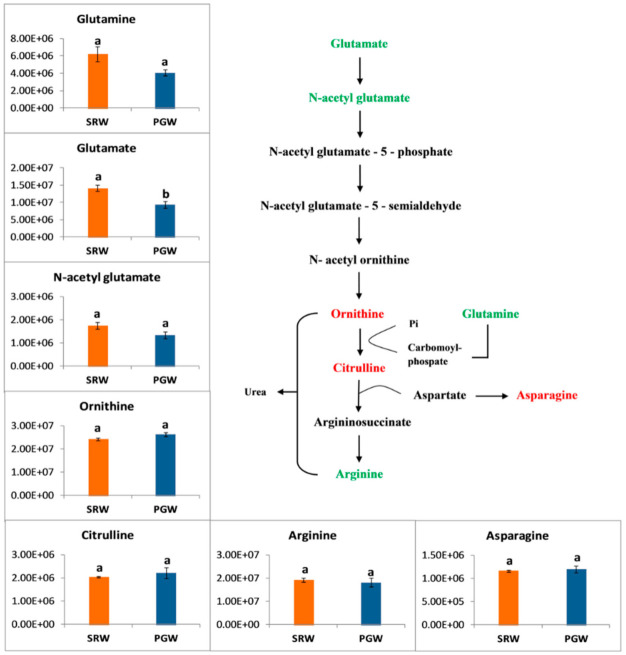
*Y*-axis shows the differences in relative contents of amino acids associated with citrulline metabolic pathway between mature fruit of pumpkin-grafted watermelon (PGW) and self-rooted watermelon (SRW). Red or green color shows the higher content of amino acids in PGW or SRW, respectively. Vertical bars represent standard error among three independent replicates. Values are the mean ± SE of three replicates. Different lowercase letters indicate significant differences at *p* < 0.05.

Nitrogen is assimilated during the arginine pathway through a series of enzymatic reactions to convert glutamate into citrulline. In the last step, citrulline changes to arginine, which is required for development and stress conditions [53]. Nitrogen is recycled and distributed in the plant via arginine metabolism. Organic nitrogen is stored and transported in the plants in the form of arginine and citrulline because of their high carbon and nitrogen ratio [54]. The catabolism of citrulline starts with acetylation of glutamate [55] followed by phosphorylation, reduction, and transamination, yielding ornithine in a cyclic pathway by several enzymes. Subsequently, citrulline and arginine are synthesized from ornithine in a linear pathway by several coordinated enzymatic reactions [55,56,57]. Several nitrogen-rich amino acids of the arginine pathway such as ornithine, citrulline, and asparagines increased by 1.09-, 1.09-, and 1.03-folds in PGW, respectively, while the amount of glutamate, glutamine, arginine, and N-acetyl glutamate decreased by 0.66-, 0.65-, 0.94-, and 0.76-folds, respectively (Figure 4). Interestingly, the grafting effect was only significant on glutamate.

### 3.6. Amino Acid Metabolism

**Figure 5:**
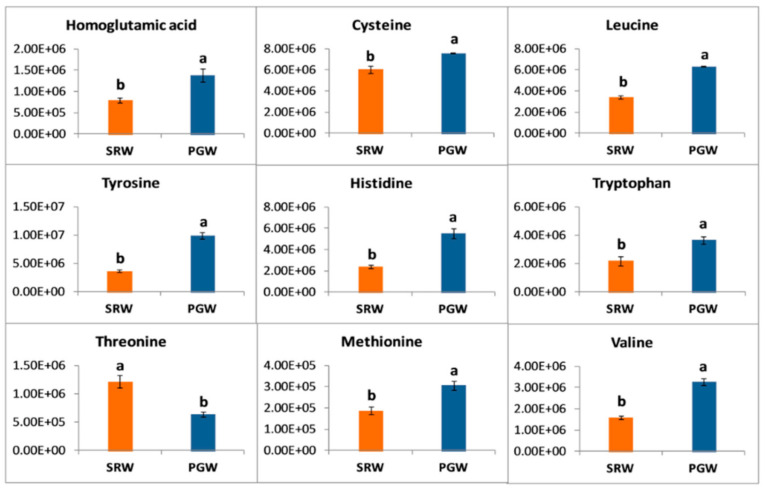
*Y*-axis shows the differences in relative contents of amino acids between mature fruit of pumpkin-grafted watermelon (PGW) and self-rooted watermelon (SRW). Vertical bars represent standard error among three independent replicates. Data are the mean ± SE of three replicates. Different lowercase letters indicate significant differences at *p* < 0.05.

In total, 51 amino acids and amino acid derivatives were detected, of which 21 amino acids and their derivatives showed significantly higher levels either in PGW or SRW. Grafting significantly elevated the contents of 18 amino acids and their derivatives (Supplementary Materials). Aromatic amino acids and their derivatives, including phenylalanine, tryptophan, tyrosine, N-hydroxy-L-tryptophan, N-acetyl-L-tyrosine, and hexose-phenylalanine, displayed significantly (*p* < 0.05) greater abundance in PGW. Alanine and gamma-aminobutyric acid involved in the GABA shunt pathway were observed to be increased by 0.80-fold in SRW (Supplementary Materials). Several other amino acids, namely homoglutamic acid, cysteine, leucine, histidine, tryptophan, and valine, exhibited significantly greater abundance in PGW. Among the sulfur-containing amino acids, methionine increased significantly (*p* < 0.05) by 1.64-folds, whereas the content of threonine decreased significantly (*p* < 0.05) by 0.5-folds in PGW (Figure 5).

### 3.7. Glutathione Metabolism

GSH is synthesized from glutamate, cysteine, and glycine by the action of two enzymes (γ-glutamylcysteine synthetase (γ-ECS) and glutathione synthase (GSHS)). γ-carboxyl group of Glu and an α-amino group of Cys are combined in the first step to form γ glutamyl-cysteine by a γ-ECS enzyme. In the next step, the GSHS enzyme carried out reaction by creating an amide bond with the α-carboxyl group of the cysteine moiety in γ-glutamylcysteine and the α-amino group of glycine to yield GSH. Then this reduced GSH is converted in oxidized GSSH form under particular circumstances such as stress or other stimuli. GSH can be directly or converted into other forms to be consumed in other chemical reactions. In the following step, cys-glyc is released, and GSH combines with a free amino acid, forming γ-glutamyl amino acid. With the removal of amino acid from γ-glutamyl amino acid 5-oxoproline is produced, and the cycle continues [58,59].

**Figure 6:**
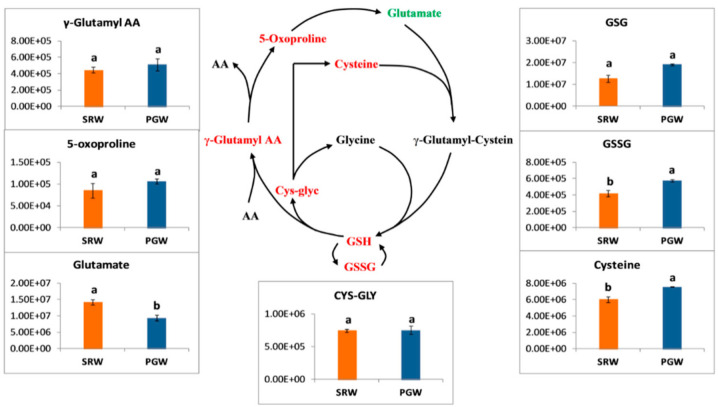
*Y*-axis shows the differences among the relative contents of intermediates of the glutathione metabolic pathway between mature fruit of pumpkin-grafted watermelon (PGW) and self-rooted watermelon (SRW). Red or green color shows the higher content of amino acids in mature fruit of PGW or SRW, respectively. Vertical bars represent standard error among three independent replicates. Data are the mean ± SE of three replicates. Different lowercase letters indicate significant differences at *p* < 0.05.

Several intermediates were detected in the metabolic pathway of glutathione and showed obvious differences between PGW and SRW. All the intermediates of this pathway were accumulated in greater abundances in PGW than SRW, including oxidized glutathione (GSSG), glutathione reduced form (GSG), 5-oxoproline, cysteine, γ-glutamyl-AA. Specifically, oxidized glutathione (GSSG) and cysteine were 1.37- and 1.26-folds (*p* < 0.05) greater in PGW than SRW. One intermediate glutamate was found significantly higher in SRW, while cys-glyc was stable in PGW and SRW (Figure 6).

### 3.8. Phenylpropanoid Metabolism

Phenylpropanoid constitutes a large group of secondary metabolites that play a central role against stresses, provide structural support to plants, and are implicated in plant survival. Range of metabolites such as flavonoids, coumarins, hydroxycinnamic acid conjugates, and lignans are produced in the metabolic pathway of phenylpropanoid. The pathway begins with phenylalanine, which undergoes a series of reactions to produce lignans, hydroxycinnamics, ferulic and sinapic acids and their corresponding esters, benzoic acid derivatives, and pigments such as chalcones, flavonoids, and anthocyanin [7,60].

**Figure 7:**
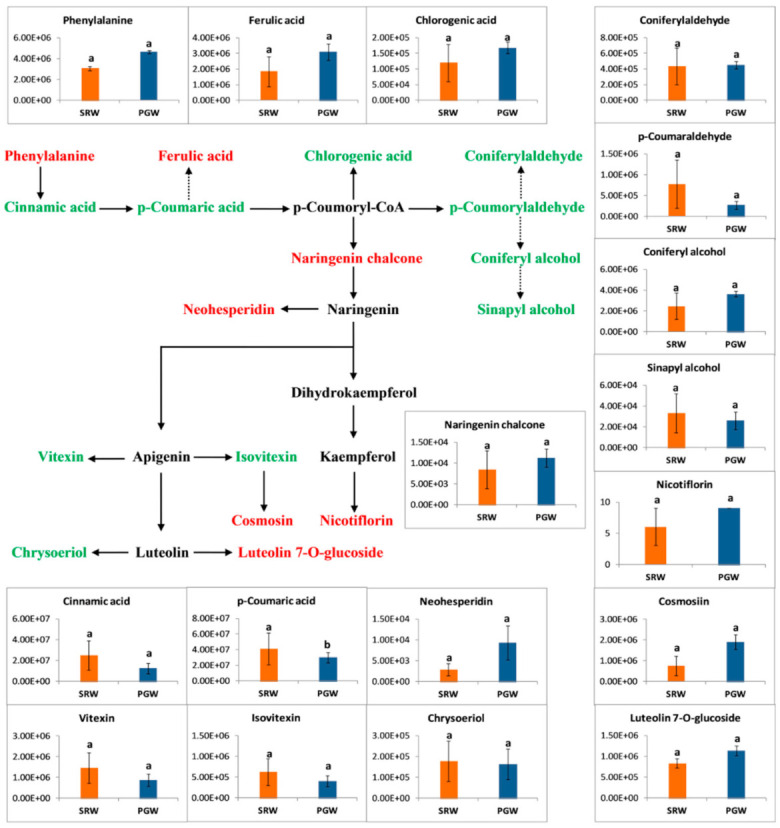
*Y*-axis shows the differences among therelative contents of intermediates of phenylpropanoid pathway between mature fruit of pumpkin-grafted watermelon (PGW) and self-rooted watermelon (SRW). Red or green color shows the higher content of amino acids in PGW or SRW, respectively. Vertical bars represent standard error among three independent replicates. Data are the mean ± SE of three replicates. Different lowercase letters indicate significant differences at *p* < 0.05.

Some secondary metabolites, such as phenols, flavonoids, flavanones, flavonols, etc. that are produced during the phenylpropanoid pathway showed slight differences with the exception of p-coumaric acid between PGW and SRW. Two flavanones (naringenin chalcone, neohesperidin), one amino acid (phenylalanine), two hydroxycinnamoyl derivatives (ferulic acid, confiryl alcohol), two flavone (luteolin 7-O-glucoside and cosmosin), one quinate derivative (chlorogenic acid), and one flavanole (nicotiflorin) were present in higher abundance in PGW as compared to SRW (Figure 7). In addition, some phenylpropanoids compounds such as cinnamic acid, p-coumaric acid, p-Coumaraldehyde, coniferylaldehyde, sinapyl alcohol, flavones-c-glycosides, vitexin, isovitexin, flavones, and chrysoeriol showed substantially higher abundances in SRW than PGW.

However, few hydroxycinnamoyl derivatives showed significant variation in PGW, of which one derivative was higher, and three were shown to have lower content in PGW. Two flavone-c-glucoside and benzoid derivatives were abundant (*p* < 0.05.) in PGW compared to SRW. Grafting caused a significant decline (*p* < 0.05) in the content of one flavone and two polyphenols (Supplementary Materials).

### 3.9. Linolenic Acid Metabolism and Fatty Acids

Linolenic acid is produced from fatty acid metabolism, which is further catalyzed into diverse classes of compounds, including ketones, alcohols, volatiles, esters, aroma compounds, aldehydes, alkenes, acids, and other compounds [61,62]. The first step is the oxidation of linolenic acid followed by dehydration of 13-hydroperoxy-octadecatrienoic acid carried out by lipoxygenases (LOXs) and allene oxide synthase, respectively [63]. The unstable epoxide generated in the above step is then cyclized stereospecifically and converted into 12-oxo-phytodienoic acid (OPDA) by allene oxide cyclase (AOC) followed by reduction and removal of side carbon chain in series of reactions that lead to the formation of jasmonic acid [64,65].

**Figure 8:**
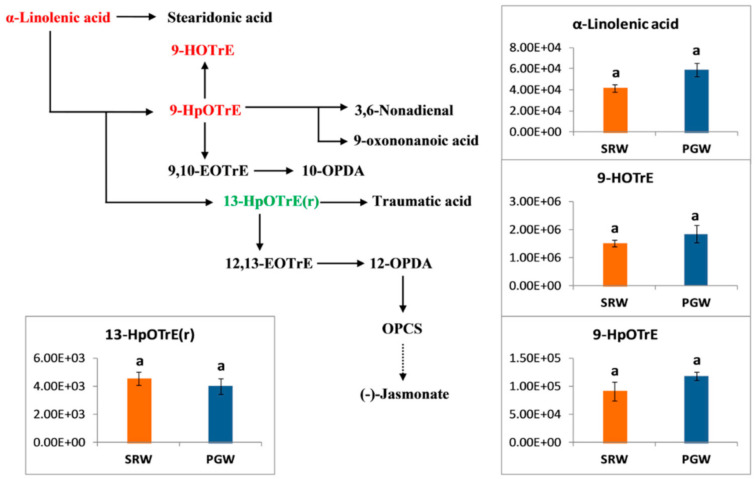
*Y*-axis shows the differences among the metabolites’ relative contents linked with a linolenic acid pathway from mature fruit of pumpkin-grafted watermelon (PGW) and self-rooted watermelon (SRW). Red or green color shows the higher content of amino acids in mature fruit of PGW or SRW, respectively. Vertical bars represent standard error among three independent replicates. Data are the mean ± SE of three replicates. Different lowercase letters indicate significant differences at *p* < 0.05.

**Figure 9:**
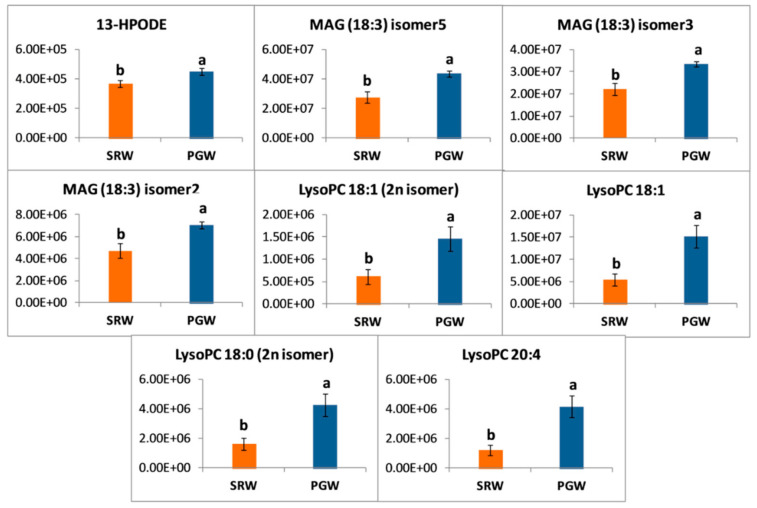
*Y*-axis shows the differences in relative contents of lipids between pumpkin-grafted watermelon (PGW) and self-rooted watermelon (SRW). Vertical bars represent standard error among three independent replicates. Data are the mean ± SE of three replicates. Different lowercase letters indicate significant differences at *p* < 0.05.

Lipids measured in this study belong to fatty acid, lipids, glycerolipids, and glycerophospholipids classes, most of the lipids accumulated in a higher amount in mature fruit of PGW than SRW. Most of the linolenic pathway intermediates showed a greater abundance in PGW than SRW, with the exception of 13-HpOTrE(r) (Figure 8). Lipids such as 13-HPODE (1.23-fold), MAG (18:3) isomer5 (1.58-fold), MAG (18:3) isomer3 (1.50-fold), MAG (18:3) isomer2 (1.49-fold), LysoPC (18:1) 2n isomer (2.39-fold), LysoPC 18:1 (2.83-fold), LysoPC 18:0 (2.59-fold), and LysoPC 20:4 (3.46-fold) were significantly abundant in mature fruit of PGW (Figure 9).

### 3.10. Secondary Metabolites

Alkaloid profiles showed obvious differences between the mature fruit of PGW and SRW. A total of seven alkaloids were detected. Of which alkaloids betain and 4beta-hydroxy-11-O-(2′-pyrolylcarboxy) epilupinine showed a significant increase of 2.32- and 2.01-folds, while but theobromine exhibited a significant decline of 0.78-fold in PGW. Most of the hormones detected showed greater abundances in PGW. Among hormones, trans-zeatin-o-glucoside was significantly abundant (1.27-folds) in PGW, while trans-zeatin-riboside and ABA showed significantly higher amounts 0.82- and 0.72-folds, respectively, in SRW.

Similarly, all the vitamins were relatively higher in PGW, while only one vitamin d-pantothenic acid (vitamin B5) was significantly higher by 2.35-folds in PGW. Metabolites that were classified as others showed striking differences between PGW and SRW. Seven metabolites accumulated in a significantly higher amount, whereas five declined in response to grafting (Supplementary Materials).

## 4. Discussion

In this study, we compared the metabolomic profile of fully mature fruit of PGW and SRW. This study provides comprehensive metabolite profiles and graft-induced changes in the mature fruit of PGW and SRW. This is the first report on large-scale metabolic profiling in grafted and self-rooted watermelon. LC-MS, as a robust tool coupled with the MIM-EPI system, is capable of identifying a large number of metabolites from one sample [66]. We have identified 339 metabolites from the fully mature watermelon fruit of PGW and SRW. Regardless of grafting, delta-aminolevulinic acid hydrochloride, sucrose, mannose-6-phosphate were dominant carbohydrates. Previously glucose, fructose, and sucrose were regarded as major sugars. The disparity in results might be due to the limited number of carbohydrates in the previous studies [9]. Carbohydrates play a key role in regulating fruit quality and taste, plant growth, energy metabolism, tolerance against biotic and abiotic stresses [67]. In grafted watermelon, carbohydrates including threose, delta-aminolevulinic acid hydrochloride, glucose-6-phophsate, and dulcitol significantly increased, while contents of galacturonic acid (gala), galacturonic acid, and glucopyranuronate inositol, sorbitol significantly declined. Sugars such as sucrose, glucose, and trehalose showed non-significant changes. Previous studies have documented the insignificant effect of similar type rootstock on glucose and sucrose sugars. [29,55,56].

In watermelon, citric acid and malic acids are considered primary organic acids [11]. Our results suggested that malic acid, azelaic acid, and H-butanoic acid ethyl ester-hexoside isomer 1 are dominant acids. However, differences in the results may be attributed to different varieties, developmental stages, analytical techniques, and environmental factors. Most importantly, earlier studies were limited in their scope and measurement of metabolites. TCA cycle generates energy as ATP, and the biosynthesis of several metabolites is directly or indirectly driven by the TCA cycle. Intermediates of the TCA cycle such as α-ketoglutaric acid, malic, and cis-aconitate and displayed higher contents in grafted watermelon fruit, whereas citrate and succinate contents were decreased. Higher malic acid and lowercitric acid contents in grafted watermelon was in agreement with previous studies [43].

Amino acids are involved in a multitude of plant physiological events besides their role in protein synthesis. Amino acids play essential roles in plant growth and development, such as redox power, resistance against stresses, and regulation of intracellular pH [68,69,70,71,72,73,74]. Several amino acids also serve as precursors for some secondary metabolites, e.g., glucosinolates [75]. Most of the amino acids accumulated at an elevated level in PGW, while a few decreased. Specifically, amino acids such as ornithine and citrulline in the arginine pathway were found in a greater amount (*p* < 0.05). In contrast, arginine was lower in abundance, showing the reduced degradation of citrulline into arginine in grafted watermelon, thus increased the abundance of citrulline [76]. Nitrogen is a crucial constituent of chlorophyll, amino acids, proteins, nucleic acid and hormones [77]. In our study, increased biosynthesis of amino acids could be attributed to increasing nitrogen uptake and nitrogen use efficiency in pumpkin-grafted watermelon, as previously reported [78]. Amino acids are developmentally regulated, and reduction in a few amino acids could explain a higher rate of protein synthesis is linked with increased consumption of amino acids, leading to the deficiency of free amino acids in grafted plants [79].

Lipids act as signaling molecules, barriers to the outside environment of the cell, and excess energy is stored in the form of lipids in plants [80]. These are also suggested to accumulate in cold stress conditions and involve in plant growth [81]. With the exception of 13-HpOTrE(r), all the intermediates of the linolenic acid pathway increased substantially in grafted fruit. Additionally, free lipids such as lycospc and its derivatives, monoacylglycerol [15], and its derivatives increase several folds in response to grafting. Substantial increase in the content of total fatty acids, total lipids, and total unsaponated lipids in the roots and leaves of watermelon and tomato after grafting was recorded [82]. Major non-enzymatic antioxidants that are associated with the glutathione metabolic pathway includes glutathione, GSH, γ-glutamyl-cysteinyl-glycine. Apart from the role in the storage and transport of reduced sulfur, GSH takes part in the detoxification of reactive oxygen species (ROS), directly or indirectly [83,84]. Glutathione enhances plant tolerance to different abiotic stresses, including salinity, drought, high and low temperature, and toxic metal stress [85,86,87,88]. Apart from glutamate, all the intermediates (GSSG), glutathione reduced form (GSG), 5-oxoproline, cysteine, and γ-glutamyl-AA. Cystein and 5-oxoproline accumulated in a higher amount in grafted watermelon. The above results indicate that pumpkin rootstock might be useful against biotic and abiotic stress, as evident from the above results. Secondary metabolites such as flavonoids, flavonols, phenolic acids, polyphenols, lignin’s, and tannins have been implicated in plant defense mechanisms against the pathogen, various stresses, UV radiation, fruit quality and graft union success [89,90,91,92,93,94,95,96,97]. Grafting show non-significant differences in phenylpropanoid and flavonoids contents, with only few up-regulated while most of them were recorded lower in grafted plants. Phenylpropanoid, namely kaempferol, was more abundant in the leaves of the grafted scion of watermelon [98].

Plant vitamins are essential for human health and play a significant role in plant metabolism and redox reaction and act as cofactors [99]. Previously, only ascorbic acid has been analyzed and observed to be increased by 40% in grafted watermelon. In our study, the fruit of grafted watermelon showed higher amounts of all vitamins, including ascorbic acid [37]. According to [45], modification of hormone status and water and nutrient uptake in grafted vegetables by specific rootstocks may lead to changes in cellular morphology, cell turgor, and cell-wall characteristics which, in turn, affect fruit firmness. In our study, IBA showed a significant increase in fruit of grafted watermelon, which may have influenced the fruit firmness and developments. Alkaloids such as nicotine reduced to one per cent when tobacco was grafted onto tomato rootstock [100]. Alkaloids are regarded as bioactive metabolites which are involved in plant environment interaction and combating against drought and control of human diseases [101]. Betain and 4beta-hydroxy-11-O-(2′-pyrolylcarboxy) epilupinine showed a relatively higher amount in grafted watermelon. Rootstock effect on scion characteristics might be due to transfer of some mobile substances, such as hormones or various RNA species, across the graft union or the differential absorption ability of rootstocks for nutrients [102]. The fact that the grafting-induced changes may be due to the process of grafting itself or the impact of heterografting to a different rootstock.

However, future studies are needed to study the regulatory mechanism responsible for changes in metabolic contents.

## 5. Conclusions

The comprehensive metabolite profiling and comparative analysis of metabolites from mature fruit of pumpkin-grafted watermelon (PGW) and self-rooted watermelon (SRW) provide novel information on metabolomics profile and changes induced by grafting. The large-scale metabolomics analysis identified 339 metabolites in PGW and SRW, and most of the metabolites were in greater abundance in PGW. These metabolites were involved in major metabolic pathways, multiple biological functions, including plant growth, phytochemicals, and stress-related metabolites. Among the secondary metabolites, hormone and vitamin B5 and alkaloids were also increased in response to grafting in PGW. Linolenic acid and its intermediates involved in the synthesis of taste, aroma, and health-related compounds were present in a greater amount in PGW. Overall, higher accumulations of metabolites in main classes of metabolic pathways represents higher plant growth and fruit quality attributes in PGW. These results confirmed the importance of using high-throughput untargeted metabolomics approach to obtain comprehensive information regarding up and down regulation of metabolites of different metabolic pathway as affected by grafting. Furthermore, additional research work is needed to understand the functioning and interaction of these pathways, gene expression, genetic and epigenetic control of these changes induced in grafted and ungrafted plants.

## Data Availability

The data presented in this study are available in article and as a Supplementary Materials.

## Supplementary Materials

The following are available online at https://www.mdpi.com/article/10.3390/biom11050628/s1, Table S1: Details of metabolites and their comparison between pumpkin-grafted and self-grafted watermelon fruit.
